# Supplementary material for: Ultra-Fine Ruthenium Oxide Quantum Dots/Reduced Graphene Oxide Composite as Electrodes for High-Performance Supercapacitors
Source: Nanomaterials (Basel). 2022 Apr 4;12(7):1210. doi: 10.3390/nano12071210 (PMC9000793; doi:10.3390/nano12071210)
Supplement: Supplementary file 1 [file nanomaterials-12-01210-s001.zip › nanomaterials-1635398-supplementary.pdf]

nanomaterials

MDPI

Supporting Information

Ultra-Fine Ruthenium Oxide Quantum Dots/Reduced Graphene Oxide Composite as Electrodes for High-Performance Supercapacitors

Jie Zhao [1], Jianmin Zhang [2,*], Hang Yin [1], Yuling Zhao [3], Guangxu Xu [1], Jinshi Yuan [1], Xiaoyao Mo [1], Jie Tang [4,*] and Fengyun Wang [1,3*]

[1] College of Physics, Qingdao University, No. 308 Ningxia Road, Qingdao 266071, China; zj120513@163.com (J.Z.); hangyinccc@gmail.com (H.Y.); tsuixgx1218@163.com (G.X.); yuanjinshi@foxmail.com (J.Y.); mxyphysics@163.com (X.M.)
[2] National Engineering Research Center for Intelligent Electrical Vehicle Power System, College of Mechanical and Electrical Engineering, Qingdao University, No. 308 Ningxia Road, Qingdao 266071, China
[3] State Key Laboratory of Bio Fibers and Eco Textiles, Qingdao University, No. 308 Ningxia Road, Qingdao 266071, China; zhaoyuling@qdu.edu.cn
[4] National Institute for Materials Science, 1-2-1 Sengen, Tsukuba 3050047, Japan
* Correspondence: zhangjm@qdu.edu.cn (J.Z.); tang.jie@nims.go.jp (J.T.); fywang@qdu.edu.cn (F.W.)

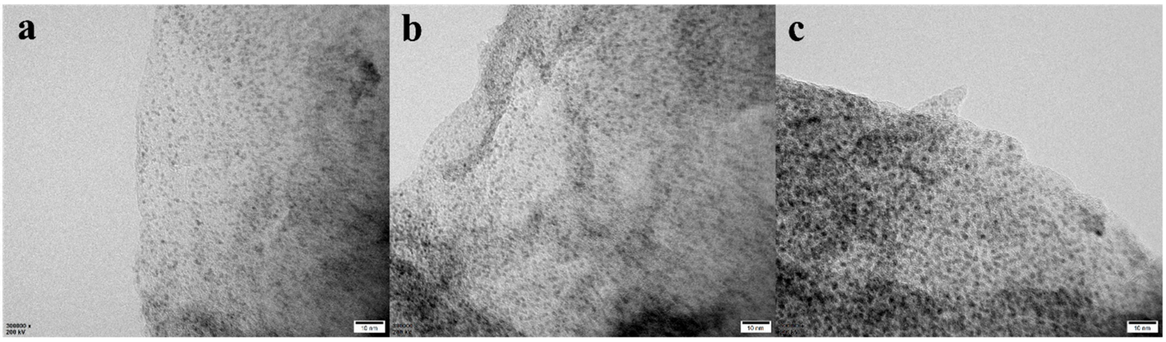

Figure S1. High magnification TEM images of (**a**) RG-1, (**b**) RG-2, (**c**) RG-3.

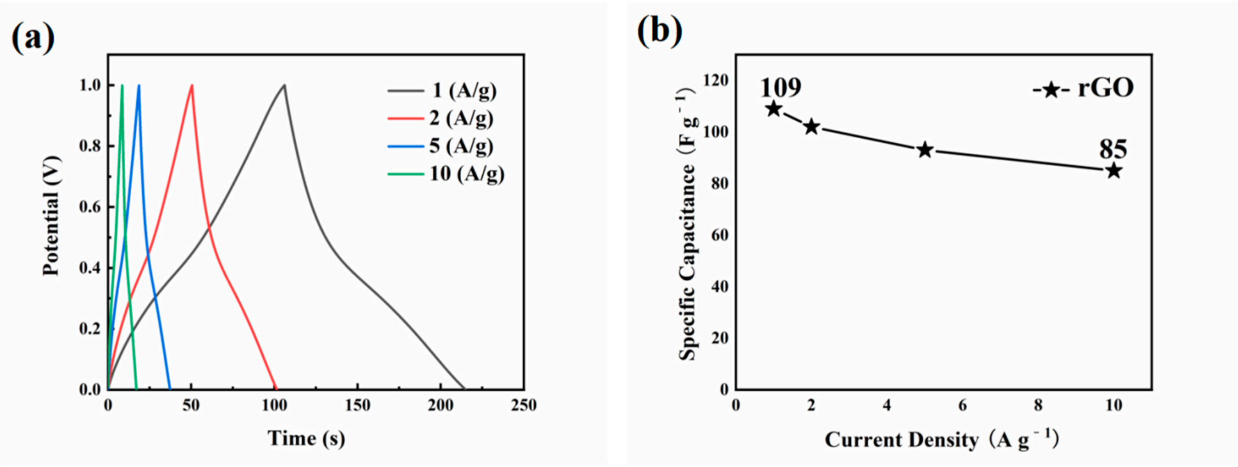

Figure S2. (**a**) charge and discharge curves of rGO. (**b**) Rate performance of rGO.

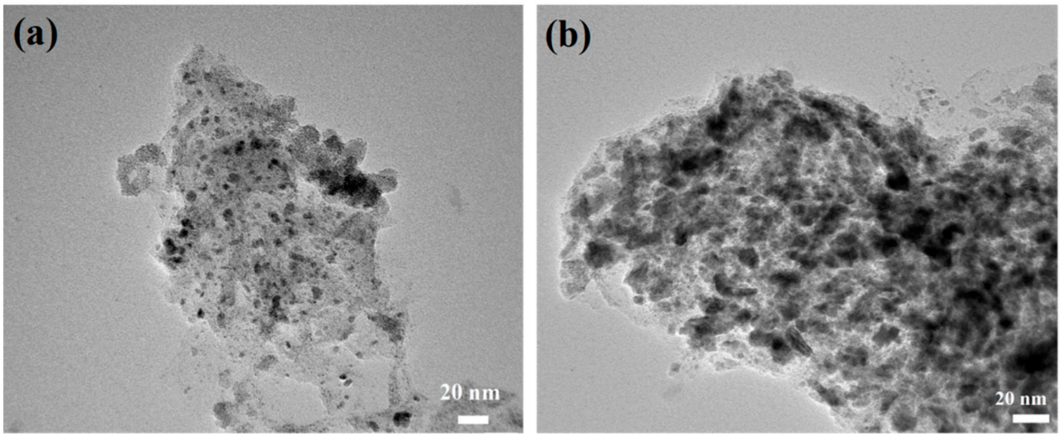

Figure S3. (**a**,**b**) TEM images of RG with the ruthenium content of 120 mg at different magnificent.
